# Supplementary material for: Kinetics of Torque Teno Virus Viral Load Is Associated with Infection and De Novo Donor Specific Antibodies in the First Year after Kidney Transplantation: A Prospective Cohort Study
Source: Viruses. 2023 Jun 28;15(7):1464. doi: 10.3390/v15071464 (PMC10384556; doi:10.3390/v15071464)
Supplement: Supplementary file 1 [file viruses-15-01464-s001.zip › viruses-2478237-supplementary.pdf]

Supplementary Table 1. Dynamics of immune and microbiologic parameters along the study period

	**PRE-TR**	**1st WEEK**	**1st MONTH**	**3rd MONTH**	**6th MONTH**	**9th MONTH**	**12th MONTH**	**p-value**
**TTV, cp/mL** Median [IQR]	1162 [0; 17983]	1185 [127; 12201]	25000 [1468; 221931]	15544548 [641703;143571751]	1308849 [33074;70735221]	182053 [4087; 5457771]	69627 [4515; 1249386]	ns Mixed-effects
**Log_{10} TTV, cp/mL** Median, IQR	3.10 [0; 4.28]	3.10 [2.10; 4.10]	4.40 [3.20; 5.38]	7.20 [5.80; 8.15]	6.10 [4.53; 7.83]	5.30 [3.60; 6.70]	4.80 [3.65; 6.10]	<0.0001 Mixed-effects
**CMV viremia** Pos, n(%) Neg, n(%)	-	2 (2.47%) 79 (97.53%)	n=80 3 (3.75%) 77 (96.25%)	3 (3.70%) 78 (96.30%)	n=80 1 (1.25%) 79 (98.75%)	3 (3.70%) 78 (96.30%)	2 (2.47%) 79 (97.53%)	ns Chi-Square
**Complement C3, mg/dL** Median, IQR	96.3 [86.5; 108.0]	100.0 [88.5; 111.0]	102.0 [91.0; 112.8]	108.0 [96.0; 120.0]	110.0 [94.25; 122.0]	109 [98; 122.5]	108.0 [97.75; 122.0]	<0.0001 Mixed effects
**Complement C4, mg/dL** Median, IQR	28.45 [23.23; 33.38]	25.00 [20.00; 31.00]	23.00 [10.00; 28.75]	26.00 [20.00; 30.00]	25.00 [21.00; 31.75]	26.00 [21.00; 31.00]	26.90 [20.05; 30.70]	<0.0001 Mixed effects
**IgG, mg/dL** Median, IQR	1180 [1018; 1298]	840 [722; 1090]	809 [703; 1025]	783 [707; 929]	887 [757; 997]	885 [782; 1070]	890 [796; 1090]	<0.0001 Mixed effects
**IgA, mg/dL** Median, IQR	249 [179; 334]	177 [125; 239]	175 [136; 246]	168 [126; 237]	175 [125; 246]	185 [133; 243]	184 [137; 245]	<0.0001 Mixed effects
**IgM, mg/dL** Median, IQR	92 [57; 113]	63 [43; 90]	74 [48; 100]	64 [40; 98]	68 [43; 101]	74 [48; 110]	79 [50; 106]	<0.0001 Mixed effects
**BKPyV viremia** Pos, n(%) Neg, n(%)	-	0 (0%) 81 (100%)	n=80 1 (1.25%) 79 (98.75%)	12 (14.81%) 69 (85.19%)	n=80 8 (10.00%) 72 (90.00%)	7 (8.64%) 74 (91.36%)	9 (1.11%) 72 (88.89%)	0.0016 Chi-Square
**JCPyV viremia** Pos, n(%) Neg, n(%)	-	0 (0%) 81 (100%)	n=80 0 (0%) 80 (100%)	2 (2.47%) 79 (97.53%)	n=80 2 (2.50%) 78 (97.50%)	3 (3.70%) 78 (96.30%)	3 (3.70%) 78 (96.30%)	0.3383 Chi-Square
**BKPyV viruria** Pos, n(%) Neg, n(%)	-	n=79 3 (3.80%) 76 (96.20%)	n=80 11 (13.75%) 69 (86.25%)	n=80 21 (26.25%) 59 (73.75%)	n=80 24 (30.00%) 56 (70.00%)	23 (28.40%) 58 (71.60%)	21 (25.93%) 60 (74.07%)	0.0001 Chi-Square
**JCPyV viruria** Pos, n(%) Neg, n(%)	-	n=79 15 (18.99%) 64 (81.01%)	n=80 15 (18.75%) 65 (81.25%)	n=80 19 (23.75%) 61 (76.25%)	n=80 25 (31.25%) 55 (68.75%)	20 (24.69%) 61 (75.31%)	26 (32.10%) 55 (67.90%)	0.2123 Chi-Square

**Creatinine, mg/dL** Median, IQR	-	1.70 [1.30; 3.00]	1.39 [1.09; 1.84]	1.29 [1.08; 1.68]	1.35 [1.16; 1.72]	1.40 [1.06; 1.72]	1.32 [1.11; 1.71]	<0.0001 Mixed effects
**eGFR, mL/min/1.73m^2** Median, IQR	-	39.0 [22.5; 58.0]	54.0 [37.3; 69.0]	56.0 [45.5; 73.5]	56.5 [43.3; 70.0]	56.0 [42.5; 73.5]	59.0 [42.0; 69.5]	<0.0001 Mixed effects
**Albumin creatinine ratio, mg/g** Median, IQR	-	-	30.1 [12.8; 86.3]	23.9 [9.7; 58.0]	27.8 [10.8; 92.6]	22.4 [9.0; 81.3]	19.9 [9.4; 64.5]	ns Mixed-effects
**Tacrolimus, µg/mL** Median, IQR	-	7.45 [5.90; 10.58]	10.30 [8.43; 11.98]	8.70 [7.25; 9.85]	7.20 [5.975; 8.700]	7.20 [5.300; 8.500]	6.20 [5.025; 7.500]	<0.0001 Mixed effects
**C-reactive protein, mg/dL** Median, IQR	-	0.800 [0.475; 1.535]	0.100 [0.100; 0.180]	0.100 [0.10; 0.40]	0.150 [0.1000; 0.4250]	0.180 [0.1000; 0.4300]	0.160 [0.1000; 0.3700]	0.0133 Mixed effects
**White blood count, Cells/uL** Median, IQR	6500 [5300; 7750]	7200 [5250; 9350]	6400 [4825; 8575]	4900 [3600; 6400]	5150 [4000; 6500]	5600 [4300; 7000]	5700 [4600; 7450]	<0.0001 Mixed effects
**Total lymphocytes, Cells/uL** Median, IQR	1563 [1364; 1691]	746 [202; 1609]	1005 [460; 1976]	1043 [605; 1722]	1081 [708; 1566]	1241 [837; 1728]	1319 [952; 1835]	0.0039 Mixed effects
**CD3+ T cells, Cells/uL** Median, IQR	1166 [827; 1372]	426 [30; 1149]	785 [245; 1570]	800 [384; 1261]	810 [474; 1265]	926 [595; 1340]	1053 [689; 1456]	0.0004 Mixed effects
**CD4+ T cells, Cells/uL** Median, IQR	770 [541; 940]	234 [10; 762]	452 [105; 1105]	465 [151; 846]	445 [167; 823]	473 [212; 826]	471 [262; 872]	0.0128 Mixed effects
**CD8+ T cells Cells/uL** Median, IQR	316 [250; 496]	123 [16; 314]	277 [118; 473]	286 [171; 459]	316 [198; 464]	379 [248; 559]	443 [320; 635]	<0.0001 Mixed effects
**CD19+ B cells Cells/uL** Median, IQR	132 [93; 193]	165 [125; 328]	194 [114; 388]	124 [66; 185]	101 [64; 165]	101 [60; 156]	109 [68; 211]	<0.0001 Mixed effects
**NK cells Cells/uL** Median, IQR	221 [140; 327]	26 [7; 106]	71 [29; 141]	115 [58; 167]	132 [86; 199]	161 [97; 243]	158 [106; 286]	<0.0001 Mixed effects

Mixed effects refers to Mixed effects analysis with the Geisser-Grenhouse correction. IQR – Interquartile range. n.s. – nonsignificant
